# Introducing cancer convergence

**DOI:** 10.1186/s41236-017-0003-x

**Published:** 2017-11-01

**Authors:** Krastan B. Blagoev, David T. Ting, Herbert Levine, Yvonne Saenger, Thea D. Tlsty, Bo Sun

**Affiliations:** 1 Johns Hopkins University, Baltimore, MD USA; 2 Massachusetts General Hospital Cancer Center, Boston, MA USA; 3 Rice University, Houston, TX USA; 4 Columbia University Medical Center, New York, NY USA; 5 University of California, San Francisco, CA USA; 6 Oregon State University, Corvallis, OR USA

One of the most remarkable expansions in complexity of life on Earth was the transition from single cell to multicellular organisms. This transition lead to cellular specialization and cooperation among cell types, including the ability to reproduce the entire organism from highly specialized single cells. Cancer is the failure of the normal cooperation among cellular components of an organ or a tissue system, and the invasion and colonization by new aberrant cells of other systems and organs. Although the specifics of each cancer vary between tissue and cell types, and often from patient to patient, these general characteristics capture the common features among all cancers at the systems level.

Despite the focus on genetic mutations and epigenetic changes as drivers of cancer in the era of molecular biology, cancer has traditionally been viewed in a more organismal fashion: as the transition from homeostasis to dysregulated behavior of a tissue that is associated with, and promoted by, altered stromal signaling, inflammatory events, failure of the immune system, miscommunications between cells and aging of tissue architecture. Often one driving factor triggers the others leading to a cascade of events that give rise to an irreversible process. This integrated failure of the system makes cancer one of the most complex yet most common diseases known to humankind. In the face of this complexity and the robustness of cancer, we require quantitative and predictive models that promote an integrated understanding of all systems involved and their interactions at many different scales from the molecular to the organismal. We believe that such understanding can be achieved only by converging all science and engineering fields on the cancer problem. Physicists, mathematicians, and engineers often look for commonalities and foundational principles among seemingly divergent natural phenomena and work to extract basic principles that are common among a class of systems. Biologists, in contrast, often focus on the detailed, distinctive behavior of a specific biological system. This dichotomy of approach has persisted and been perpetuated despite modern biology’s increasing embrace of computational analysis and quantitative models which requires the need for developing a system to handle the flood of data, for quantitating the distinctive behaviors and evaluating their statistical significance. The quantitative sciences offer a powerful set of tools in the quest for common fundamental principles and connections among seemingly disparate biological phenomena. The synthesis of these two approaches will be essential for solving the cancer problem.

The new Springer Nature journal *Cancer Convergence* is an open access platform for quantitative research on tissue homeostasis and how cancer is a departure from it, with the aim of helping to accelerate the development of new experimental and theoretical approaches understanding cancer and opening new therapeutic avenues. In striving to be the leading journal in which to discuss the details and validation of new, multi- and interdisciplinary approaches, *Cancer Convergence* serves as an avenue for biologists, mathematicians, physicists, chemists, engineers, as well as data and computer scientists to publish boundary-pushing papers on a diversity of formal methodologies, conceptual insights and results from their laboratories. Uncovering the principles of physics and biology that underlie the origin and progression of cancer and govern its response to treatment will be accomplished through a convergence of physical sciences and engineering on the analysis of the many components of biological systems affected by the disease.

Complete understanding of cancer is impossible without stepping beyond the current habit of describing mechanisms without embedding them in conceptual frameworks of formal first principles that underlie processes taking place at different spatial and temporal scales. A quest for universal principles will entail the use of the language of mathematics that is at the core of “predictive models”. Equally as important will be the development of conceptual breakthroughs that illuminate the complex systems biology of cancer even if such insights still lack formalization in mathematical terms.

*Cancer Convergence* welcomes all high-quality studies that use a quantitative or first-principles approach to advance the understanding of cancer. We welcome submissions on diverse topics including, but not limited to: fundamental physics such as forces and energy changes in cancer; new insights on the genomic and epigenetics of cancers; cellular and molecular events that maintain or disrupt homeostasis; mathematical modeling of the treatment and response to therapies; machine learning for analysis of genomic and epigenetic data; and the development of imaging techniques and analysis of imaging data. We also welcome papers that address tissue homeostasis in in vivo and in vitro systems as well as aging and its relation to cancer. And finally, we would like *Cancer Convergence* to be the journal of choice for papers devoted to the explicit presentation of technical methods that are used in other studies. Often technical details of a study embody the actual originality of a study and are critical for the new insights gained. These technical details are of primary interest to the technical community but are often buried in supplements, and thus do not bestow the appropriate credit on the scientists who developed the methods.

